# Celecoxib attenuates hepatosteatosis by impairing de novo lipogenesis via Akt‐dependent lipogenic pathway

**DOI:** 10.1111/jcmm.17435

**Published:** 2022-06-17

**Authors:** Cong Zhang, Yuzhen Lu, Yingying Song, Liang Chen, Junjie Hu, Yan Meng, Xin Chen, Shan Li, Guohua Zheng, Zhenpeng Qiu

**Affiliations:** ^1^ College of Pharmacy Hubei University of Chinese Medicine Wuhan People's Republic of China; ^2^ Hubei Key Laboratory of Resources and Chemistry of Chinese Medicine Hubei University of Chinese Medicine Wuhan People's Republic of China; ^3^ Hubei Key Laboratory of Wudang Local Chinese Medicine Research Hubei University of Medicine Shiyan People's Republic of China; ^4^ Department of Biochemistry Institute of Basic Medical Sciences, Hubei University of Medicine Shiyan People's Republic of China; ^5^ Key Laboratory of Chinese Medicine Resource and Compound Prescription Ministry of Education, Hubei University of Chinese Medicine Wuhan People's Republic of China

**Keywords:** Akt, celecoxib, de novo lipogenesis, hepatic steatosis, non‐alcoholic fatty liver disease

## Abstract

Mounting evidence indicates that hepatic de novo lipogenesis is a common abnormality in non‐alcoholic fatty liver disease (NAFLD) patients. We investigated whether a selective COX‐2 inhibitor, celecoxib, alleviates hepatic steatosis by targeting an Akt‐driven lipogenic pathway. We estimated the efficacy of celecoxib in a novel Akt‐driven NAFLD mouse model established via hydrodynamic transfection of activated forms of AKT and in fructose‐fed NAFLD mice that exhibited increased insulin‐independent hepatic lipogenesis. AKT‐transfected and insulin‐stimulated human hepatoma cells were used for the in vitro experiments. Haematoxylin and eosin staining, immunohistochemistry and immunoblotting were performed for mechanistic studies. The results revealed that celecoxib ameliorated hepatic steatosis in the AKT‐triggered NAFLD mice. Mechanistically, celecoxib effectively suppressed AKT/mTORC1 signalling and its downstream lipogenic cascade in the Akt‐driven NAFLD mice and in vitro. Furthermore, celecoxib had limited efficacy in alleviating hepatic lipid accumulation and showed no influence on lipogenic proteins associated with hepatic lipogenesis in fructose‐administered mice. This study suggests that celecoxib may be favourable for the treatment of NAFLD, especially in the subset with Akt‐triggered hepatic lipogenesis.

## 1 INTRODUCTION

Non‐alcoholic fatty liver disease (NAFLD) is an increasing public health concern defined as a continuum of liver disorders ranging from pure fatty infiltration to a potentially progressive form of non‐alcoholic steatohepatitis (NASH) occurring in individuals without alcohol abuse.^1^, ^2^ Epidemiological data indicate a dramatic increase in the morbidity and mortality of NAFLD‐related advanced chronic liver diseases worldwide, such as fibrosis, cirrhosis and hepatocellular carcinoma (HCC) in some fatal cases.^3^, ^4^ In Western countries, paralleling the mounting prevalence of obesity, dyslipidaemia, diabetes mellitus and insulin resistance, NAFLD/NASH is considered a component of metabolic syndrome. However, this condition also occurs at a lower body mass index (BMI) in less‐developed regions of Asia and manifests in patients without a previous diagnosis of diabetes or insulin resistance.^2^ Despite the variable epidemiological patterns of NAFLD worldwide, excessive intrahepatic triglyceride (TG) accumulation, namely predominant macrovesicular steatosis, is a general prerequisite needed to support the development of this disease, rendering hepatocytes more susceptible to a set of lipotoxic inducers and pro‐inflammatory cytokines and thus the condition progresses to advanced liver diseases.^5^

Unconstrained lipogenesis has been linked to NAFLD at the molecular level, reflected by the coordinated elevated expressions or activities of lipogenic enzymes transcriptionally activated by sterol regulatory element‐binding protein (SREBP), liver X receptor (LXR) and carbohydrate responsive element‐binding protein (ChREBP) in hepatocytes.^6^ Concerning the potential sources of fatty acids that facilitate hepatic steatosis, Donnelly et al. proposed that while 60% of hepatic TG in NAFLD patients originates from plasma non‐esterified fatty acids (NEFA) and 10% from dietary fatty acids, nearly 30% of the total liver TG may be produced through de novo lipogenesis,^7^ indicating a growing contribution of the activated lipogenic cascade in hepatic TG storage in this phenotype, which could be promising metabolic targets for the treatment of NAFLD.

Consistent with a pro‐inflammatory role in chronic liver diseases,^8^ the temporal profile of enhanced cyclooxygenase 2 (COX‐2) expression paralleled the progression of NAFLD in rodent models,^9^ implying that COX‐2 and other pro‐inflammatory factors involved could be therapeutic targets for single agents or combination regimens intended to achieve disease remission and reversal. Although it has previously been demonstrated that celecoxib, a COX‐2 selective non‐steroidal anti‐inflammatory drug (NSAID) widely prescribed for relieving arthritis‐related pain, alleviates high‐fat diet (HFD)‐induced hepatic steatosis^10^ and metabolic steatohepatitis in murine models,^9^, ^11^ the precise mechanisms by which suppression of de novo lipogenesis contributes to its role in the lipogenic NAFLD phenotype need to be better defined. In this study, we performed interventional studies to elucidate whether celecoxib ameliorates hepatosteatosis induced by enhanced de novo lipogenesis in mice with hepatic overexpression of activated v‐akt murine thymoma viral oncogene homologue (AKT),^12^ as well as in fructose‐fed mice featuring steatosis of hepatocytes driven by an Akt‐independent lipogenic cascade.^13^ Our data indicate that celecoxib efficiently ameliorated hepatic steatosis by repressing hepatic de novo lipogenesis in an Akt‐dependent manner.

## 2 MATERIALS AND METHODS

### 2.1 Constructs and reagents

The plasmids used for hydrodynamic injection, including pT3‐EF1α‐HA‐myr‐AKT and pCMV‐sleeping beauty transposase (SB), were gifts from Dr. Xin Chen of the University of California, San Francisco, CA, USA. Before mouse injection or transient transfection, the plasmids were prepared using an E.Z.N.A.® Endo‐Free Plasmid Maxi Kit (Omega Bio‐Tek). Celecoxib (CAS # 169590–42‐5) and fructose (purity ≥99%) were purchased from Aladdin Reagent Co., Ltd. Bovine insulin was purchased from Sigma‐Aldrich. A Cell Counting Kit‐8™ was obtained from Dojindo Laboratories for the colorimetric cell viability assay. The antibodies used in this study and their applications are listed in Table S1. All other chemicals were of analytical grade.

### 2.2 Hydrodynamic injection and mouse treatment

Female wild‐type (WT) FVB/N mice were purchased from Charles River Laboratories (Beijing, China). The hydrodynamic injection was performed as previously described^12^ for inducing hepatic steatosis in 6‐8‐week‐old mice. In brief, a normal saline solution (2 ml) containing 20 μg of plasmids encoding AKT (pT3‐EF1α‐HA‐myr‐AKT) along with 1.6 μg of plasmids of SB (pCMV‐SB) was injected into the caudal veins of mice within 7 s. To evaluate the therapeutic efficacy of celecoxib in vivo, celecoxib (50 and 100 mg/kg) or vehicle was administered via gavage once daily, 3 days post‐transfection for five consecutive weeks. At the end of the experimental period, the mice were euthanized by exsanguination under isoflurane‐anaesthetised conditions. Blood samples were collected and immediately centrifuged to obtain the serum. Liver samples were stored at −80°C until further investigation. Immunohistochemistry was performed to determine the efficiency of hydrodynamic injection of AKT using an anti‐total Akt antibody (Figure S1). All animal experiments were performed according to protocols approved by the Committee for Animal Research at the Hubei University of Chinese Medicine. CELE‐L and CELE‐H represent intragastric administration of celecoxib at low (50 mg/kg) and high (100 mg/kg) doses, respectively.

### 2.3 Animal model of fructose‐induced steatosis and celecoxib treatment

A fructose‐induced hepatic steatosis animal model was established as previously described.^14^ Briefly, female FVB/N mice (6–8 weeks old) fed standard chow had free access to plain tap water (WT control) or a 30% fructose solution for 8 weeks. The fructose‐fed mice were simultaneously orally administered with celecoxib (50 and 100 mg/kg) or vehicle. No obvious weight loss or food intake decrease occurred in the celecoxib‐received cohorts (data not shown). At the end of the experimental period, the mice were euthanized after anesthetization, as mentioned above. Liver tissues were collected for further investigation.

### 2.4 Haematoxylin–eosin (H&E) and oil red O (ORO) staining

Formalin (4%) fixed liver tissues were embedded in paraffin and sectioned for further detection. The sections were dewaxed with xylene, rehydrated with ethanol at decreasing concentrations and then stained with H&E reagent. For ORO staining, frozen sections of the liver specimens were incubated with the ORO solution, rinsed with 60% isopropanol and counterstained with haematoxylin. H&E and ORO staining were visualized and evaluated using a light microscope (Olympus IX 73 DP80). The pathological grading and staging of NAFLD mice were scored according to the system reported by *Kleiner* et al.^15^

### 2.5 Biochemical and enzymatic assays

The triglyceride (TG), alanine aminotransferase (ALT) and aspartate aminotransferase (AST) levels were analysed using standard methods in accordance with the manufacturer's protocols (Jiancheng Bioengineering Institute, Nanjing, Jiangsu, China).

### 2.6 Western blotting, immunohistochemistry (IHC) and real‐time quantitative polymerase chain reaction (RT‐qPCR)

For Western blot analysis, aliquots of 40 μg were denatured by boiling in Tris‐Glycine SDS Sample Buffer, separated by SDS‐PAGE and transferred onto polyvinylidene fluoride (PVDF) membranes by electroblotting. The membranes were blocked with 5% nonfat dried milk in Tris‐buffered saline containing 0.05% Tween 20 for 1 h and probed with the specific primary antibodies. β‐actin was used as a loading control. Each primary antibody was followed by incubation with an anti‐rabbit/mouse IgG/HRP (secondary) antibody. Protein bands were visualized using SuperSignal® West Pico PLUS chemiluminescent substrate (Thermo Fisher Scientific) and images were obtained using a gel documentation system (G: BOX Chemi XRQ) with high‐resolution camera and GeneSys (v1.5.5.0) imaging software.

Immunohistochemical staining of mouse liver tissue specimens was performed on 4% formalin‐fixed, paraffin‐embedded sections. For antigen retrieval, slides were microwaved in 10 mM citrate buffer (pH 6.0) for 10 min. The immunoreactivity was visualized using an SP Rabbit & Mouse HRP Kit (CWBIO) according to the manufacturer's protocol, with DAB as chromogen. The slides were counterstained with haematoxylin.

An RT‐qPCR assay was performed using FastStart Universal SYBR Green Master Mix (Roche) on a CFX96™ RT‐qPCR cycler (Bio‐Rad). Cycling conditions were as follows: 2 min of denaturation at 95°C followed by 40 cycles of 10 s at 95°C, 30 s at 62°C and 15 s at 72°C. The housekeeping gene β‐actin was used as an internal control for quantitative analysis. The identified primers for DNA amplification are listed in Table S2.

### 2.7 Cell culture and treatment

Human hepatoma HepG2 and Huh‐7 cells were purchased from the Shanghai Cell Bank of the Chinese Academy of Sciences and maintained as monolayer cultures in Dulbecco's modified Eagle's medium supplemented with 10% foetal bovine serum (FBS) (Gibco) and antibiotics at 37 °C under an atmosphere of 5% CO_2_. Cell line authentication was performed by STR profiling before the initiation of this study. All in vitro experiments were repeated at least three times.

The pT3‐EF1α‐HA‐myr‐AKT vectors were transiently transfected into hepatoma cells for 24 h using Lipofectamine™ 2000 Transfection Reagent (Invitrogen) following the manufacturer's instructions. The exogenously AKT‐expressed hepatoma cells were further administered with celecoxib (10–50 μM) for 24 h.

For establishing insulin‐stimulated in vitro cell models, hepatoma HepG2 or Huh‐7 cells at 80% confluence were maintained in serum‐free media overnight prior to celecoxib treatment for 30 min. The celecoxib‐incubated cells were stimulated with 100 nM insulin for 30 min by adding insulin to the celecoxib‐containing culture medium. For chronic insulin treatment, hepatoma cells were incubated in DMEM containing 0.1% FBS with 100 nM insulin for 48 h. The medium was refreshed every 12 h. The chronically insulin‐stimulated cells were further incubated either in the absence or the presence of celecoxib at the indicated doses for 24 h. Cell lysates were harvested using M‐PER® Mammalian Protein Extraction Reagent (Thermo Fisher Scientific) supplemented with protease/phosphatase inhibitors and analysed by immunoblotting.

### 2.8 Statistical analysis

Data analysis was performed using Prism 7.0 (GraphPad Software Inc.). Data obtained from at least three independent experiments are shown as the mean ± standard deviation (SD) values. Comparisons between two groups and among three or more groups were achieved using the two‐tailed unpaired *t*‐test or analysis of variance (anova). Values of *p* < 0.05 indicated statistical significance.

## 3 RESULTS

### 3.1 Celecoxib ameliorates Akt‐driven hepatic steatosis in mice

**FIGURE 1:**
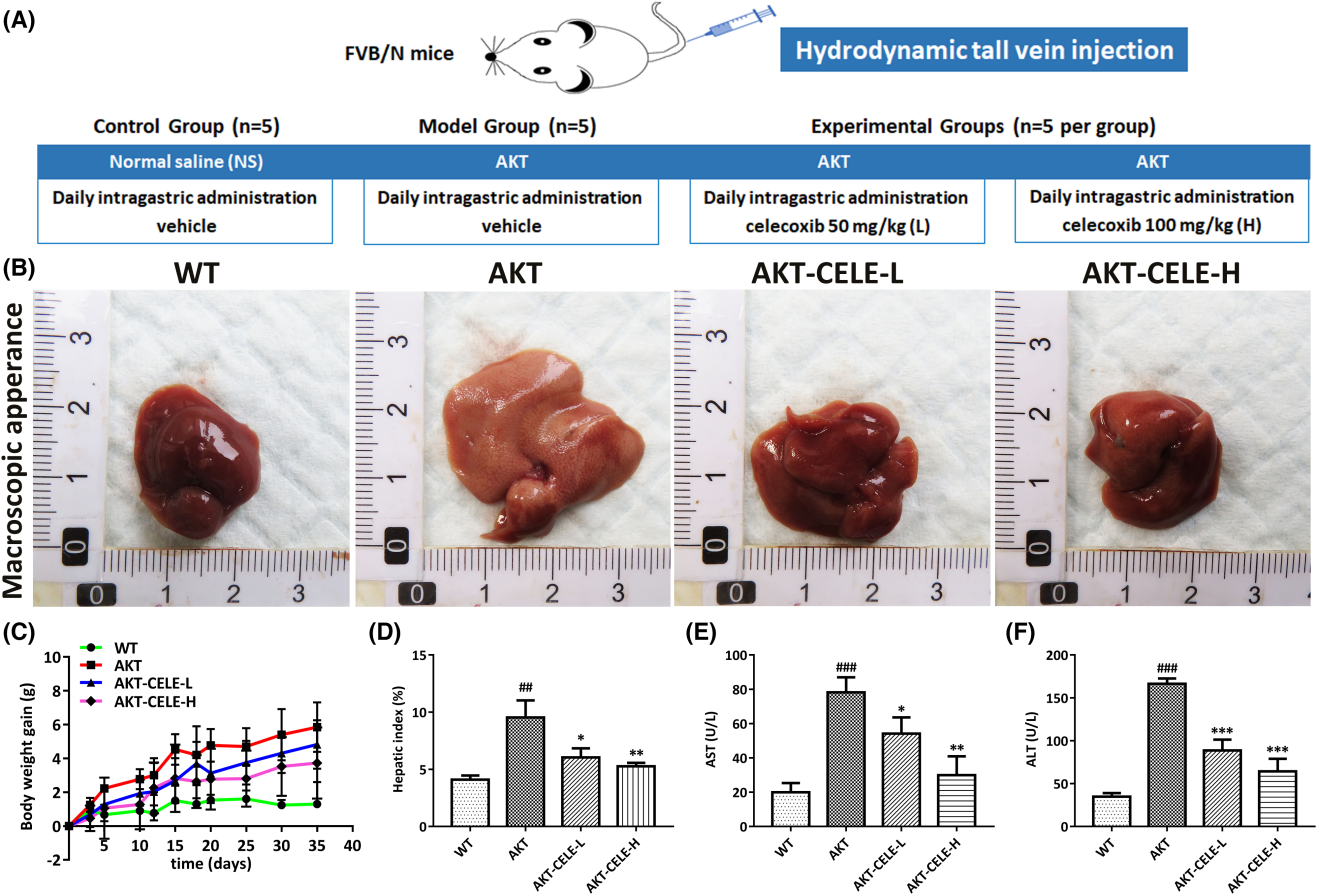
Celecoxib delays AKT‐driven hepatic steatosis in mice. (A) Study design. (B) Macroscopic appearance of liver tissues from the WT cohort and the AKT mice with intragastric administration of either vehicle or celecoxib, respectively. (C) The effects of celecoxib administered by gavage on body weight gain in the AKT mice. (D–F) Mice treated with celecoxib display a decline in liver/body ratios (D), alanine transaminase (ALT) (E) and activities in serum (F) aspartate transaminase (AST). Mean ± SD, *n* = 5. ##*p* < 0.01, ###*p* < 0.001 versus the WT group; *p < 0.05, **p < 0.01, ***p < 0.001 versus the AKT group. Abbreviations: CELE, celecoxib; CELE‐L and CELE‐H represent intragastric administration of celecoxib at low (50 mg/kg) and high (100 mg/kg) doses, respectively

**FIGURE 2:**
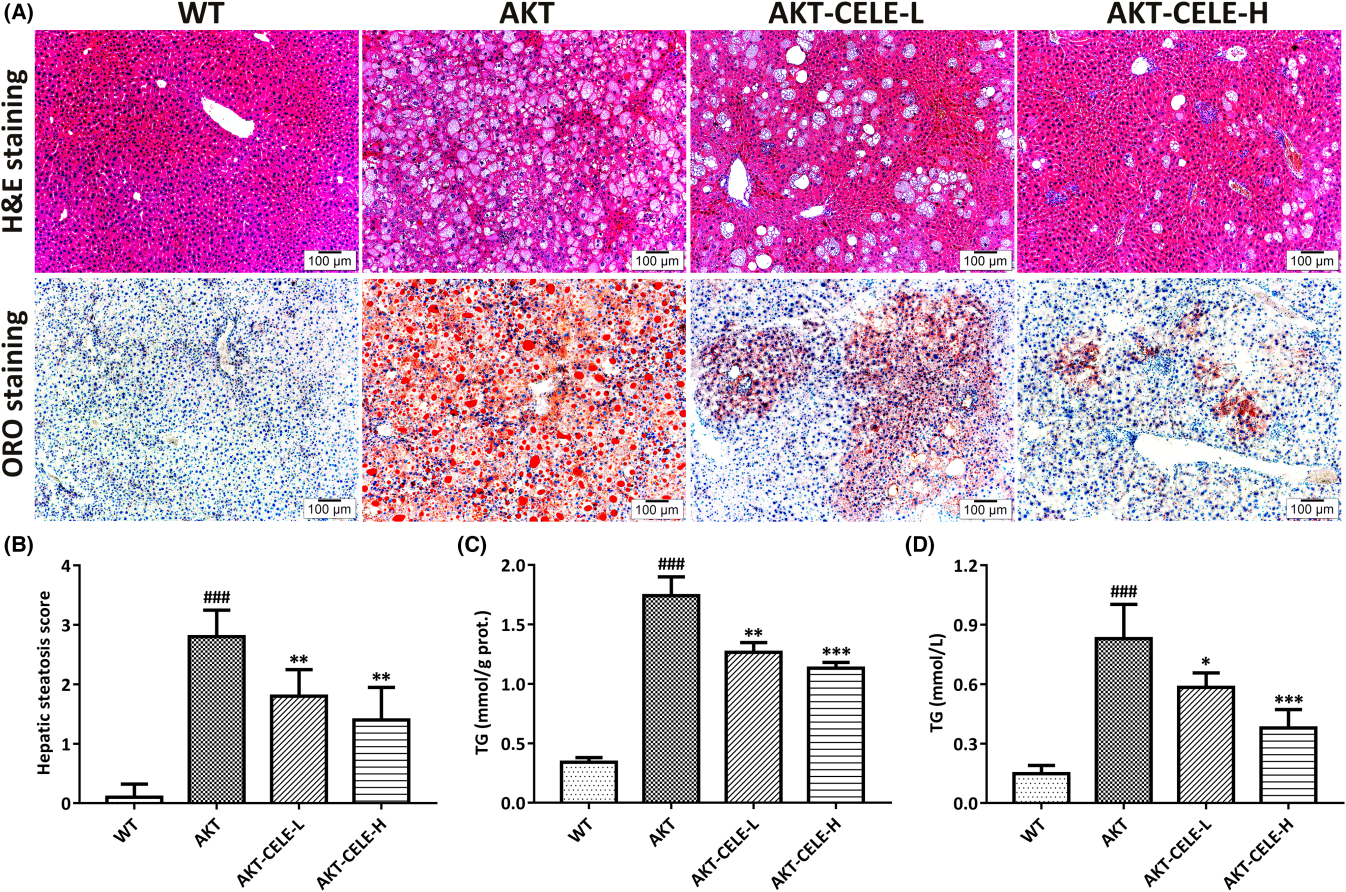
Celecoxib alleviates hepatic steatosis and lipid accumulation in AKT mice (A) Hematoxylin‐eosin (H&E) and Oil Red O (ORO) staining of the AKT‐driven mice with celecoxib (CELE) administration for five weeks. Original magnification: 100×; Scale bar: 100 μm. (B–D) Mice subjected to CELE exhibited a decline in hepatic steatosis score (B) accompanied by a reduction in hepatic and serum triglyceride (TG) levels (C, D). Mean ± SD, *n* = 5. ###*p* < 0.001 versus the WT group; *p < 0.05, **p < 0.01, ***p < 0.001 versus the AKT group. Abbreviations: CELE, celecoxib; protein, Prot. CELE‐L and CELE‐H represent intragastric administration of celecoxib at low (50 mg/kg) and high (100 mg/kg) doses, respectively

Intrahepatic overexpression of Akt contributes to aberrant de novo lipogenesis, leading to severe hepatic steatosis in mice within 5 weeks post hydrodynamic injection.^12^, ^16^ Here, we investigated the therapeutic effect of celecoxib in antagonizing lipogenesis‐induced NAFLD progression in AKT‐injected mice. We began celecoxib therapy at the initiation of hepatic steatosis, namely 3 days post vector injection. The mice were orally administered the vehicle or celecoxib by gavage (Figure 1A). Macroscopically, a uniformly pale fatty liver and hepatomegaly occurred 5 weeks post‐injection in the vehicle‐treated cohort, while celecoxib treated mice (50 mg/kg) displayed a healthy normal liver size and reduced weight gain and liver/body weight ratios (Figure 1B–D). Meanwhile, serum aminotransferase (ALT/AST) was significantly elevated in the AKT‐injected mice compared with those in WT mice, whereas celecoxib inhibited the increase in these biochemical indicators (Figure 1E,F) of liver dysfunction.^17^ Moreover, lipid accumulation in hepatic intracellular vacuoles, occupying more than half of the liver parenchyma in the vehicle‐treated mice, was alleviated by celecoxib administration, as shown by histological evidence and steatosis score intensity analysis (Figure 2A,B). Consistently, AKT‐injected mice concomitantly administered with CELE displayed reduced hepatic and serum triglyceride levels compared with controls (Figure 2C,D). Overall, our data suggest that celecoxib effectively prevents the development of Akt‐triggered hepatic steatosis in mice.

### 3.2 Celecoxib attenuates inflammatory response in livers of AKT mice

**FIGURE 3:**
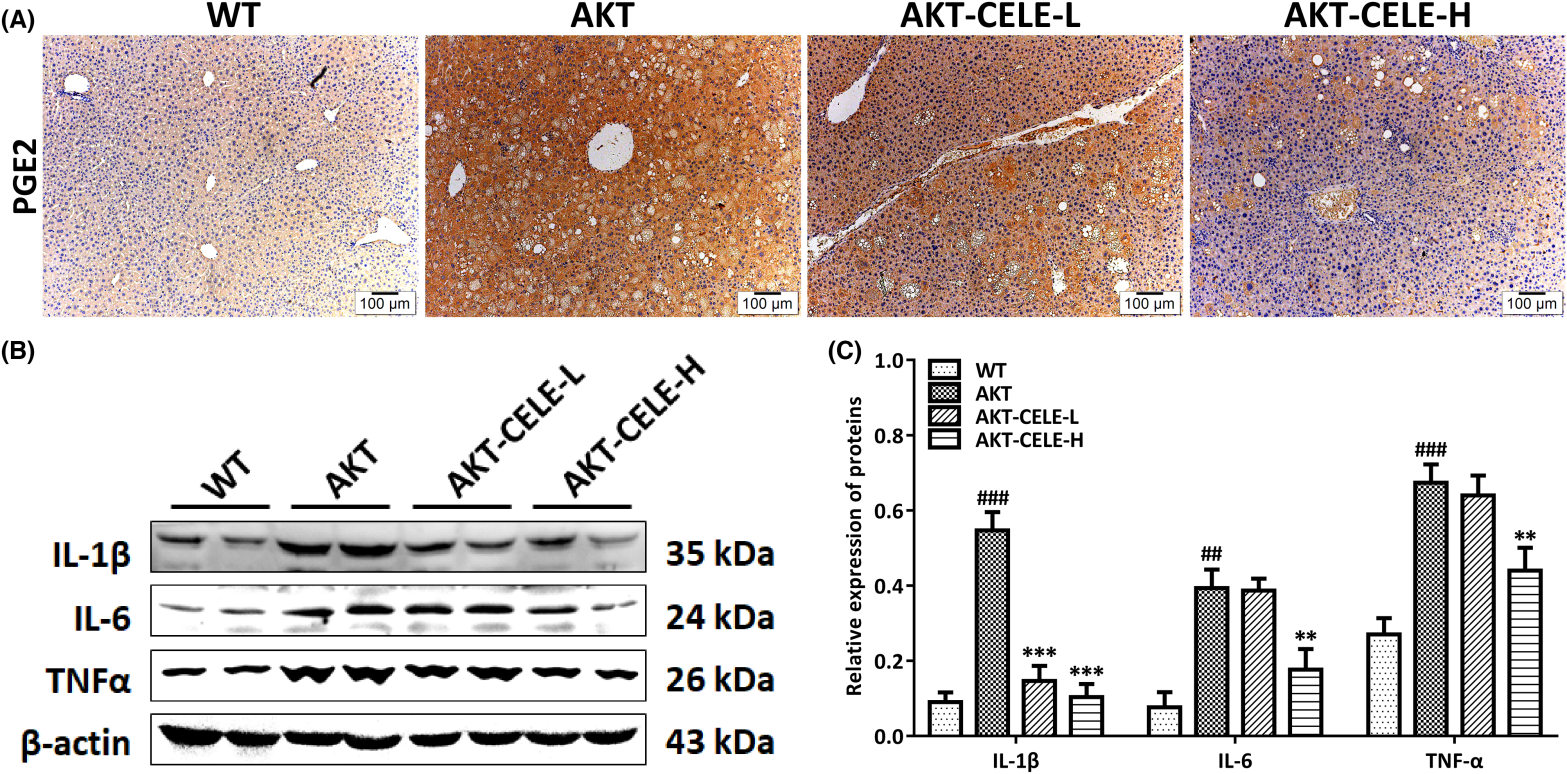
Celecoxib impairs COX‐2‐related inflammatory responses simultaneously in the livers of AKT mice. (A) PGE2 staining in liver tissues from the WT cohort and the AKT mice with either vehicle or celecoxib intragastric administration. Celecoxib lightens the staining (yellow‐brown) of PGE2 localized in the cytoplasm. Original magnification: 100×; scale bar: 100 μm. (B) Western blotting was performed to analyze the protein expression of IL‐1β, IL‐6, and TNF‐α. (C) Histograms represent the protein expression of IL‐1β, IL‐6, and TNF‐α quantified through the western blotting optical analysis shown in (B). β‐actin was used as an internal reference. Immunoreactive bands (including those with white backgrounds) were visualized in a gel documentation system (G: BOX Chemi XRQ, Syngene) without further modification. Quantified data are presented as mean ± SD, *n* = 5. ##*p* < 0.01, ###*p* < 0.001 versus the WT group; **p < 0.01, ***p < 0.001 versus the AKT cohort. Celecoxib is defined as CELE. CELE‐L and CELE‐H represent intragastric administration of celecoxib at low (50 mg/kg) and high (100 mg/kg) doses, respectively

Next, as the local inflammatory response is crucial in augmenting hepatic steatosis in NAFLD/NASH,^18^ we determined the protein levels of inflammatory cytokines facilitating hepatic lipid accumulation in these mice. The results suggested that the macroscopic or histopathological alterations in the AKT mouse livers were paralleled by the overproduction of prostaglandin E2 (PGE2) (Figure 3A). As expected, celecoxib significantly repressed the production of PGE2 in the livers of AKT mice (Figure 3A). Western blotting analysis revealed that celecoxib treatment reduced the release of IL‐1β/6 and TNF‐α in livers compared with untreated AKT mice (Figure 3B,C). Thus, the data indicate that celecoxib restrained pro‐inflammatory responses during Akt‐evoked hepatic steatosis in mice.

### 3.3 Celecoxib suppresses lipid metabolic reprogramming by impairing Akt phosphorylation in the liver of AKT mice

**FIGURE 4:**
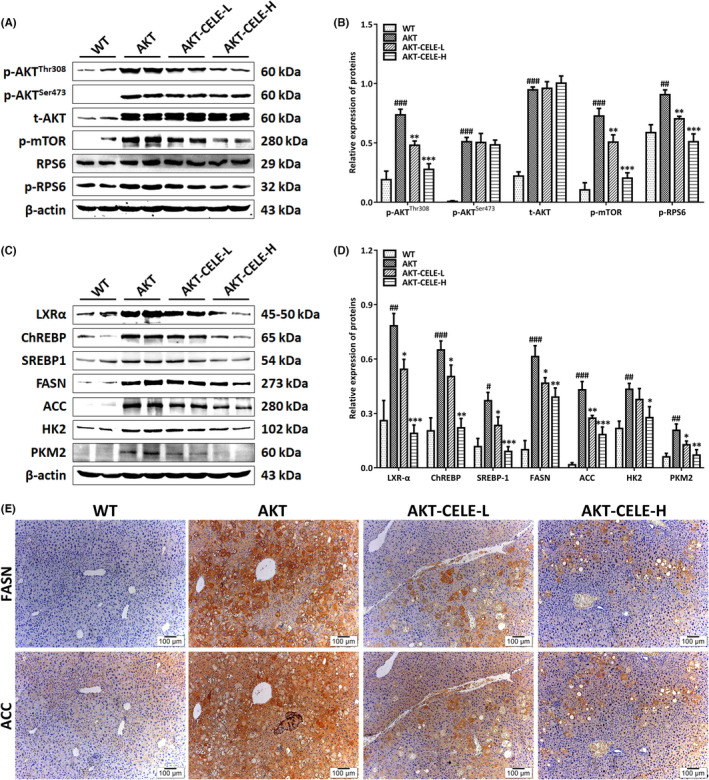
Celecoxib suppresses an AKT‐driven lipogenic pathway in livers of AKT mice. Western blotting was performed to analyze the Akt/mTORC1 signaling (A) and lipogenic transcriptional proteins (C) in liver tissues from the WT cohort and the AKT mice intragastrically administered either vehicle or celecoxib. Histograms in (B) and (D) represent the expression of key components in the Akt/mTORC1 signaling and its downstream lipogenic pathway quantified through the western blotting optical analysis shown in (A) and (C), respectively. β‐actin was used as an internal reference. Immunoreactive bands (including those with white backgrounds) were visualized in a gel documentation system (G: BOX Chemi XRQ, Syngene) without further modification. Quantified data are presented as mean ± S.D., *n* = 5. #*p* < 0.05, ##*p* < 0.01, ###*p* < 0.001 versus the WT group; *p < 0.05, **p < 0.01, ***p < 0.001 versus the AKT group. (E) Immunohistochemical staining (yellow‐brown localized in the cytoplasm) of FASN and ACC in the liver of wild‐type (WT) mice or the AKT mice in the absence or presence of celecoxib. Original magnification: 100×; scale bar: 100 μm. Abbreviations: CELE, celecoxib; t, total; p, phosphorylated. CELE‐L and CELE‐H represent intragastric administration of celecoxib at low (50 mg/kg) and high (100 mg/kg) doses, respectively

To further illuminate the molecular mechanisms underlying the therapeutic efficacy of celecoxib, we evaluated the protein expression of major effectors mediating Akt‐triggered de novo lipogenesis in the vehicle or celecoxib‐treated liver samples. Progressive induction of total and phosphorylated Akt occurred in the steatotic livers from AKT mice compared with that in control livers, as detected by immunoblotting (Figure 4A,B). Hepatic Akt, mammalian target of rapamycin (mTOR) and ribosomal protein S6 (RPS6) activation were paralleled by a pronounced upregulation of key lipogenic transcription factors (ChREBP, LXRα, SREBP1) and enzymes (fatty acid synthase, FASN; acetyl‐CoA carboxylase, ACC) at both transcriptional (Figure S2) and post‐transcriptional (Figure 4A,B) levels in the AKT mice. Notably, we observed that celecoxib suppressed phosphorylation of Akt at Thr308, with no influence on Akt Ser473 phosphorylation (Figure 4A,B). Moreover, celecoxib treatment inhibited the phosphorylation of mTOR and RPS6 (Figure 4A,B). Notably, hepatic FASN and ACC reduction occurred at the mRNA (Figure S2) and protein (Figure 4C,D) levels in the liver of celecoxib‐treated cohorts, presumably due to the impaired expression of lipogenic transcription factors compared with that in the vehicle‐treated group, as assessed by immunoblotting (Figure 4C,D). Immunohistochemical staining also provided consistent evidence (Figure 4E).

**FIGURE 5:**
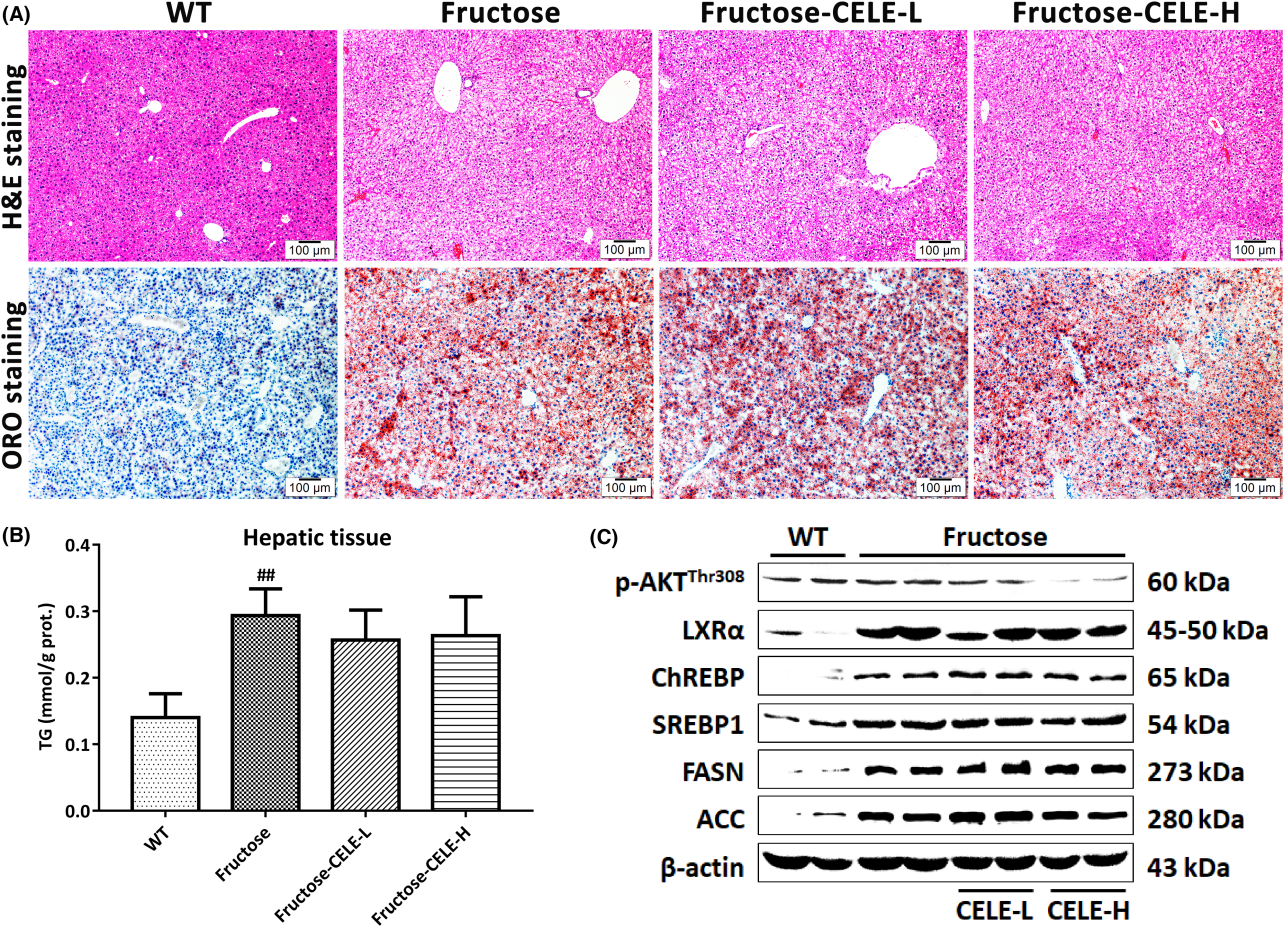
Celecoxib fails to ameliorate fructose‐induced steatosis of hepatocytes in mice. Female FVB/N mice (*n* = 6 per group, 6–8 weeks old) fed with normal chow had free access to plain tap water (WT control) or 30% fructose solution for eight weeks. The fructose‐fed mice were simultaneously orally treated with celecoxib at the same doses administered in the AKT mice (50 and 100 mg/kg). (A) H&E and ORO staining of liver tissues from the WT cohort and the fructose‐induced mice with intragastric administration of either vehicle or celecoxib. (B) TG contents of the mouse liver tissues. (C) Immunoblotting analysis of p‐AKT (Thr308) and key lipogenic proteins in the mouse liver tissues. β‐actin was used as an internal reference. Celecoxib had no influence on the protein expression of lipogenic transcriptional factors and enzymes in liver tissues from fructose‐fed mice. Abbreviations: CELE, celecoxib; Prot., protein; p, phosphorylated. CELE‐L and CELE‐H represent intragastric administration of celecoxib at low (50 mg/kg) and high (100 mg/kg) doses, respectively

Next, we investigated whether celecoxib affected the aforementioned lipogenic targets in the livers of fructose‐fed mice, a preclinical non‐alcoholic fatty liver model featuring enhanced de novo lipogenesis stimulated in an insulin/Akt‐independent manner.^13^ The results indicated that celecoxib had no effect on fructose‐induced steatosis of hepatocytes or the elevation of key lipogenic factors (Figure 5). Together, the data suggest that celecoxib alleviates liver TG accumulation by impairing the Akt‐driven lipogenic pathway.

### 3.4 Celecoxib impairs the Akt‐induced lipogenic response in vitro

**FIGURE 6:**
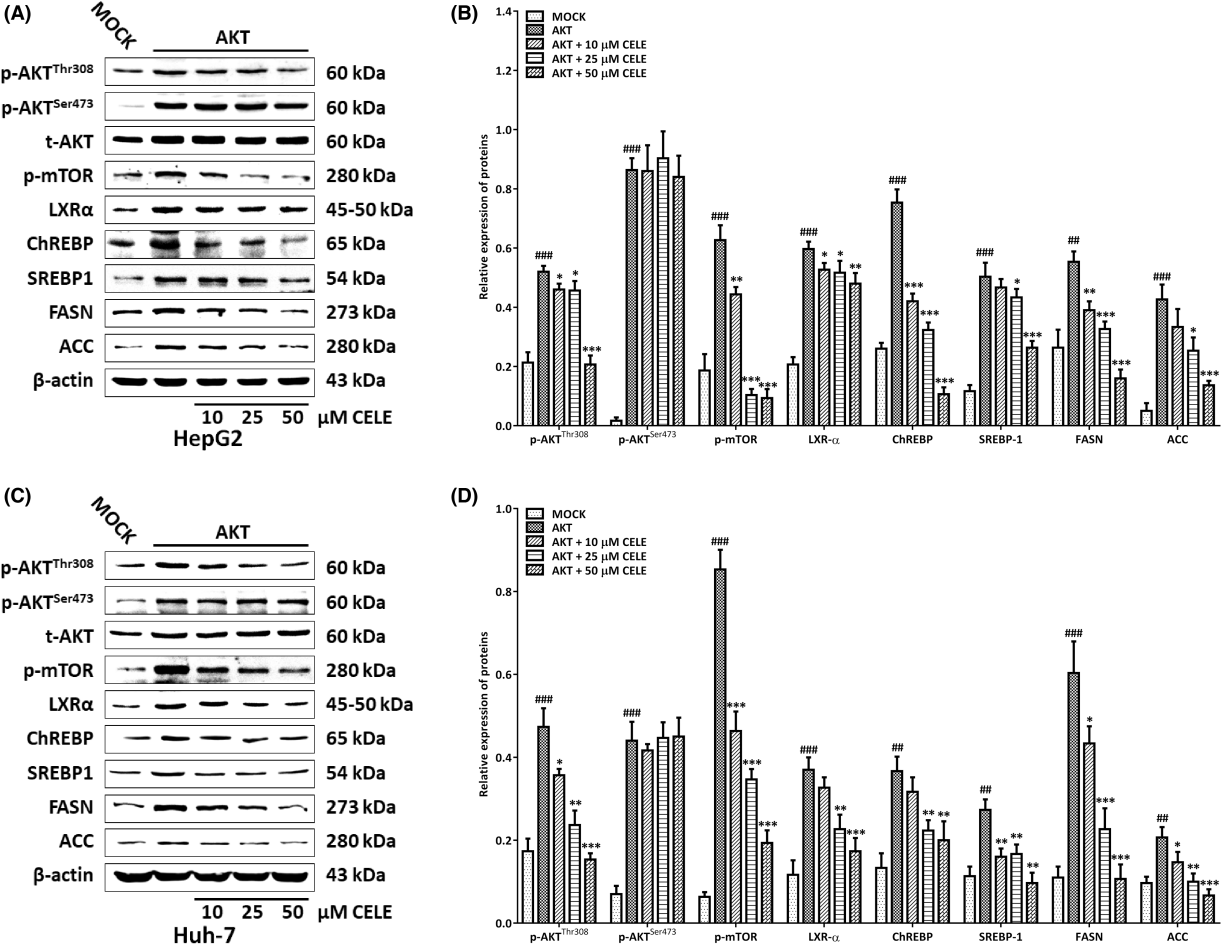
Celecoxib inactivates the Akt/mTORC1 signaling and represses its downstream lipogenic proteins in vitro. For AKT transient transfection, hepatoma HepG2 and HuH‐7 cells were seeded on plates and transfected with AKT constructs for 24 h as described in the Methods section. The transfected cells were further incubated with celecoxib (10–50 μM). Western blotting (A, C) and quantitative analysis (B, D) were performed to analyze the expressions of key components in the Akt/mTORC1 signaling and its downstream lipogenic pathway. β‐actin was used as an internal reference. Immunoreactive bands (including those with white backgrounds) were visualized in a gel documentation system (G: BOX Chemi XRQ, Syngene) without further modification. Quantified data are presented as mean ± SD, ##*p* < 0.01, ###*p* < 0.001 versus the MOCK group; *p < 0.05, **p < 0.01, ***p < 0.001 versus the AKT‐transfected group. Abbreviations: CELE, celecoxib; t, total; p, phosphorylated

To further substantiate the mechanistic findings in vivo, we investigated whether celecoxib could affect the Akt‐dependent lipogenic effectors in cell‐based studies. All subsequent experiments were conducted using 10–50 μM celecoxib as previously described.^19^ In the untreated AKT‐transfected HepG2 cells, the activated Akt/mTOR signalling induced by forced overexpression of Akt was paralleled by the enhanced expression of the lipogenic proteins (Figure 6A,B). Consistent with the in vivo findings, celecoxib suppressed the protein expressions of p‐AKT (Thr308), p‐mTOR, LXRα, ChREBP, SREBP1, FASN and ACC in AKT‐transfected HepG2 cells, while it had no influence on AKT Ser473 phosphorylation, as indicated by the equal expression levels of p‐AKT (Ser473) in the vehicle or celecoxib‐treated groups (Figure 6A,B). Similar results were obtained when Akt‐overexpressed Huh‐7 cells were subjected to celecoxib administration (Figure 6C,D).

**FIGURE 7:**
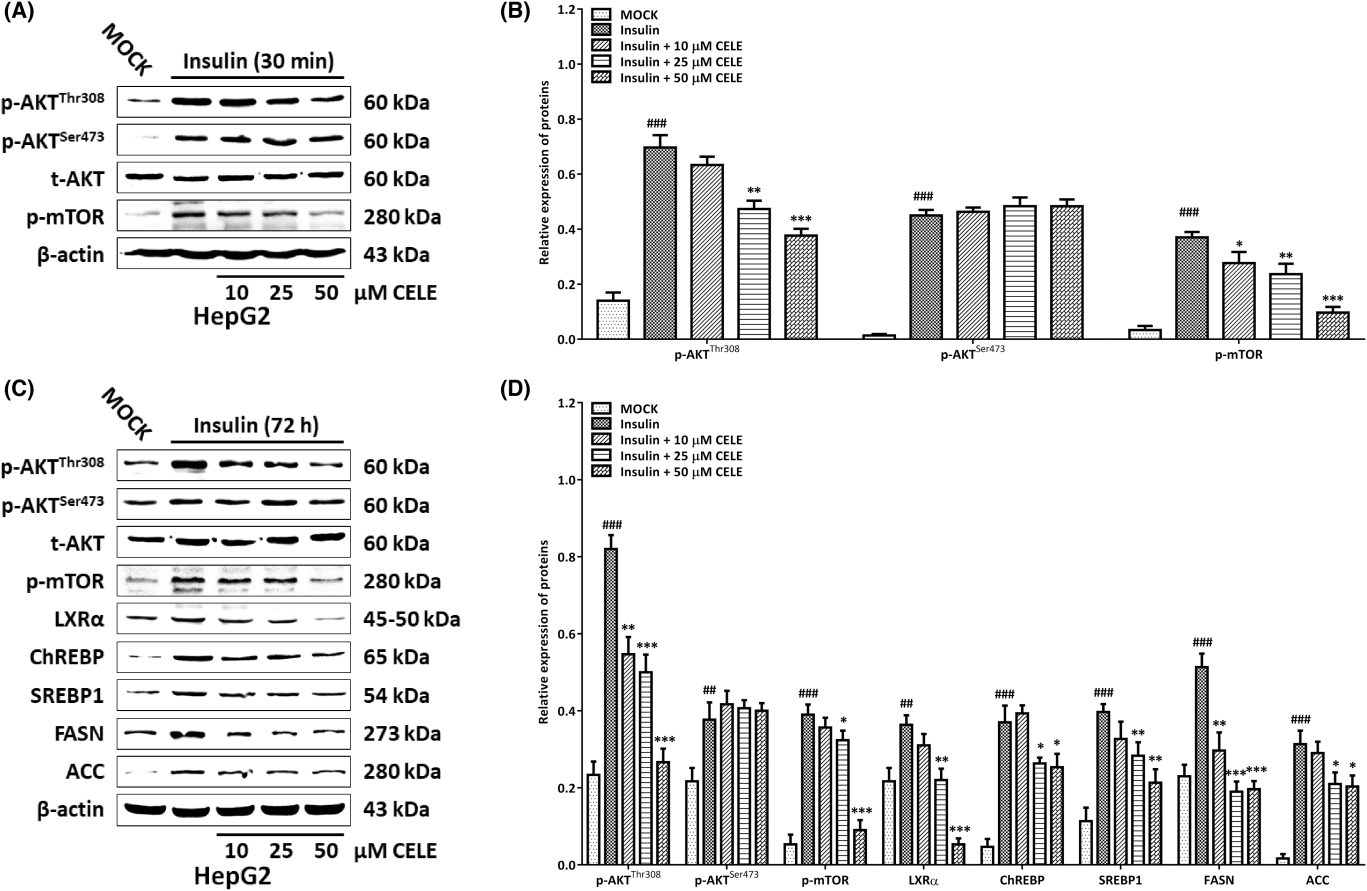
Celecoxib represses insulin‐driven Akt/mTORC1 signaling and its downstream lipogenic pathway in vitro. (A) Immunoblotting analysis of Akt and mTOR phosphorylation in HepG2 cells upon incubation with insulin for 30 min in the presence (10–50 μM) and absence of celecoxib. (C) Immunoblotting analysis of the phosphorylation of Akt/mTOR and expression of key lipogenic proteins in HepG2 cells with chronic incubation (72 h) with insulin (100 nM), in the presence (10–50 μM) and absence of celecoxib. Histograms in (B) and (D) represent the expression of key components in the Akt/mTORC1‐dependent lipogenic cascade quantified using western blotting optical analysis shown in (A) and (C), respectively. Immunoreactive bands (including those with white backgrounds) were visualized in a gel documentation system (G: BOX Chemi XRQ, Syngene) without further modification. Quantified data are presented as mean ± SD, ##*p* < 0.01, ###*p* < 0.001 versus the MOCK group; *p < 0.05, **p < 0.01, ***p < 0.001 versus the insulin‐incubated group. Abbreviations: CELE, celecoxib; t, total; p, phosphorylated

Insulin‐stimulated Akt activation is responsible for hepatic de novo lipogenesis.^20^ We further assessed whether celecoxib could counteract insulin‐mediated Akt phosphorylation and its downstream lipogenic protein expression in vitro. To achieve this goal, serum‐starved cells were preincubated in the presence or absence of celecoxib prior to acute stimulation (30 min) with insulin and then, the levels of Akt and mTOR phosphorylation were assessed. Western blotting demonstrated that insulin‐stimulated mTOR phosphorylation was absent upon preincubation with celecoxib, an effect associated with reduced insulin‐induced AKT Thr308 phosphorylation (Figure 7A,B), yet celecoxib had no influence on insulin‐stimulated AKT Ser473 phosphorylation in HepG2 cells. Next, we tested the effect of celecoxib on the lipogenic response in chronically insulin‐treated HepG2 cells.^21^ Consistently, upon insulin incubation for 72 h, HepG2 cells displayed elevated levels of p‐AKT and p‐mTOR. (Figure 7C,D). Notably, celecoxib significantly repressed the Akt/mTOR signalling and reduced the protein expression of LXRα, ChREBP and SREBP1, thus restraining the induction of FASN and ACC (Figure 7C,D). Overall, these data indicate that celecoxib effectively suppresses insulin/Akt‐stimulated lipogenic reprogramming by repressing AKT Thr308 phosphorylation.

## 4 DISCUSSION

An expanded pool of hepatic lipids results from an imbalance between dietary uptake, the influx of fatty acids from peripheral tissues, local biosynthesis, transport and egress. Notably, increased de novo lipogenesis has emerged as a major contributor to the accumulation of cytoplasmic lipid droplets in NAFLD.^22^ Here, we demonstrate that a COX‐2 inhibitor, celecoxib, alleviates hepatic steatosis and the inflammatory response in a non‐nutrient‐induced mouse model of NAFLD featuring increased de novo lipogenesis via the hydrodynamic transfection of the activated form of AKT, also known as protein kinase B (PKB), a conserved threonine/serine kinase for genome stability, nutrient availability and cellular energy metabolism. Mechanistically, celecoxib efficiently inhibited Akt/mTORC1 signalling in hydrodynamically AKT‐injected mice and in vitro, subsequently suppressing the lipogenic pathway that potentiates lipid metabolic reprogramming in NAFLD. The present study represents the first direct confirmation that celecoxib is efficacious in lipogenesis‐related NAFLD by suppressing the Akt‐dependent enhancement of hepatic de novo lipogenesis.

Genetic, chemical/toxin‐mediated and dietary/nutrient murine models of NAFLD provide critical information for illuminating the pathogenetic mechanisms of NAFLD and determining the therapeutic efficacy of potential agents.^23^ Further, it is essential to use these models that conform to the purpose of the NAFLD investigations.^24^ Hepatic steatosis is the most notable histological feature of NAFLD.^25^ Although previous studies have suggested that celecoxib attenuates liver steatosis and inflammation in rodents fed a high‐energy diet,^10^, ^11^, ^26^ it is tricky to elucidate whether it can reduce hepatocellular lipid accumulation by targeting de novo lipogenesis in these models because the steatosis indiscriminately results from excessive fatty acid uptake, lipolysis in adipocytes and hepatic lipogenesis. Instead, enhanced intrahepatic expression of activated AKT independently elicits local lipogenesis and elevates the expression of lipogenic enzymes, facilitating macrovesicular steatosis in mice.^12^, ^16^ Here, we provide solid evidence that celecoxib reduces lipogenesis‐triggered hepatic steatosis in AKT‐transfected mice, accompanied by the suppression of hepatocellular inflammatory responses (TNF‐α, IL‐6 and IL‐1β). Indeed, TNF‐α is a potential therapeutic target in NAFLD. Moreover, systemic and local metabolic dysfunction facilitates lipid accumulation of hepatocytes, enhancing hepatocellular TNF‐α production and activating its downstream kinases. Furthermore, TNF‐α‐mediated inflammatory signalling pathways impair insulin receptor signal transduction, resulting in insulin resistance, which is crucial for NAFLD progression.^27^ Thus, it would be important to further investigate whether celecoxib ameliorates hepatic insulin resistance by regulating TNF‐α signalling in a NAFLD mouse model induced by Akt‐driven lipogenesis and characterized by insulin resistance. Interestingly, emerging evidence has also suggested that inflammation shows pleiotropic efficacy in modulating the progression of NASH via its adverse and favourable effects.^28^ For instance, the participation of IL‐6 in NAFLD remains controversial. A recent clinical trial showed a positive correlation between hepatic IL‐6 expression and the severity of NAFLD.^29^ IL‐6 signalling, on the contrary, was found to play a protective role against the development of hepatic steatosis in methionine‐choline deficient diet‐fed mice, whereas it may exacerbate liver inflammation.^30^ Clearly, additional experiments are required to clarify the reciprocity of these incompatible functions in lipogenesis‐related NAFLD and the therapeutic strategies of celecoxib or other NSAIDs.

In accordance with previous studies elucidating the molecular mechanisms by which celecoxib obstructs the evolution of liver cancer,^19^, ^31^, ^32^ we observed that celecoxib suppressed the Akt/mTORC1 signalling that plays a decisive role in governing de novo lipogenesis by potentiating the efficiency of lipogenic gene networks in the Akt mice. Furthermore, although insulin‐driven PI3K/Akt signalling stimulates the hepatic SREBP1c transcription factor to orchestrate hepatic lipogenesis,^20^, ^33^, ^34^ we cannot fully conclude that celecoxib impairs de novo lipogenesis in an insulin/Akt‐dependent manner based on the inhibitory effects of celecoxib on the lipogenic transcription factor levels (LXRα, ChREBP and SREBP1) and key enzymes (FASN and ACC) in mice and in vitro. Mechanistically, we further demonstrated that celecoxib abrogated the activation of Akt/mTOR signalling in insulin‐stimulated hepatoma cells, followed by the reduction of downstream lipogenic proteins at the post‐transcriptional level in chronically insulin‐treated cohorts.

We also investigated the efficacy of celecoxib in fructose‐fed mice. Fructose consumption is a risk factor for NAFLD. In hepatocytes, fructose is preferentially converted into fructose‐1‐phosphate (F1P) by fructokinase in an insulin‐independent manner. Meanwhile, lipogenic cascades are simulated by a high flux of fructose to the liver after an acute fructose load as the lipogenic precursors derived from fructose carbons accumulate since the transformation of F1P can bypass glycosylation of phosphofructokinase 1. Additionally, chronic fructose intake also directly activates lipogenic transcription factors. Thus, fructose insulin‐independently serves as both a substrate and an inductor of de novo lipogenesis, facilitating liver steatosis.^35^, ^36^ However, in contrast to the investigation in the AKT‐transfected mice, no changes were detected in the hepatic expression of lipogenic transcription factors and enzymes in celecoxib‐treated fructose‐fed mice, paralleled by an equivalent degree of steatosis histologically observed in the liver tissues of fructose‐fed and celecoxib‐treated cohorts. Indeed, the manipulation of gluconeogenesis and lipogenesis of hepatocytes is influenced under pathological conditions of insulin resistance in metabolic syndrome. In some NAFLD patients with insulin resistance, insulin fails to restrain hepatic glucose production, whereas increased hepatic lipogenesis is sustained, namely selective insulin resistance, exacerbating both the hyperglycaemic and hyperlipidaemic situations.^37^ Thus, we hypothesize that celecoxib may effectively delay the progression of hepatic steatosis to advanced liver diseases in individuals with hepatic metabolic alterations induced by the insulin‐driven activation of PI3K/AKT/mTORC1 signalling in hepatocytes. In addition, the aberrant regulation of hepatic glucose production induces diabetes, insulin resistance and NAFLD by impairing AKT Ser473 phosphorylation in the liver and peripheral adipose tissues.^38^ Meanwhile, restoring appropriate Akt activity alleviates hepatic steatosis.^39^ In this regard, the celecoxib‐induced inhibition of AKT Thr308 phosphorylation might be indifferent if it occurred in NAFLD manifested in diabetes mellitus featuring insulin resistance. Previously, a study reported that celecoxib improved insulin sensitivity and showed efficacy in NAFLD mice with insulin resistance.^11^ However, the precise mechanisms by which celecoxib ameliorates NAFLD/NASH by affecting Akt activity under insulin‐resistant conditions remain unclear. Intriguingly, it has also been reported that celecoxib directly elevated hepatic AKT Ser473 phosphorylation and thus alleviated hepatic lipid accumulation and injury in mice fed a methionine choline‐deficient (MCD) diet.^40^ In contrast, our data show that it has no effect on this residue, as demonstrated by the equivalent levels of phosphorylated AKT (Ser473) in the AKT mice or cell‐based studies, regardless of the presence or absence of celecoxib. These distinct patterns of efficacy may be partly explained by the distinct function of local lipogenesis in different NAFLD phenotypes. Unlike the findings observed in those dietary/nutrient murine models of NAFLD, the present study suggests that celecoxib directly weakens de novo lipogenesis, which plays a decisive role in Akt‐driven hepatic steatosis, by inhibiting the lipogenic response in an Akt‐dependent manner. Clearly, it is imperative to further investigate the underlying mechanism by which celecoxib differentially regulates NAFLD development by utilizing animal models that closely resemble all aspects of the intricate etiopathogenesis and histological features of human NAFLD at different stages.

Taken together, this study indicates that celecoxib suppresses de novo lipogenesis and alleviates hepatic steatosis by inactivating Akt/mTORC1 signalling and by suppressing its downstream lipogenic factors. Our in vivo and cell‐based data support further investigations employing celecoxib and other NSAIDs for NAFLD prevention and therapy. Additional studies using precise NAFLD models might be significant. Furthermore, combined therapy of celecoxib with other metabolic agents (e.g., AMP‐activated protein kinase activator metformin) should be evaluated to confirm the validity of COX‐2 inhibitors in the treatment of NAFLD.

## AUTHOR CONTRIBUTIONS

**Cong Zhang:** Conceptualization (equal); data curation (equal); investigation (equal); methodology (equal). **Yuzhen Lu:** Conceptualization (equal); data curation (equal); investigation (equal); methodology (equal). **Yingying Song:** Data curation (supporting); investigation (equal). **Liang Chen:** Formal analysis (lead). **Junjie Hu:** Software (equal); visualization (equal). **Yan Meng:** Software (equal); visualization (equal). **Xin Chen:** Software (equal); visualization (equal). **Shan Li:** Resources (equal). **Guohua Zheng:** Conceptualization (equal); project administration (lead). **Zhenpeng Qiu:** Funding acquisition (lead); resources (equal); writing – review and editing (lead).

## CONFLICT OF INTEREST

The authors confirm that there are no conflicts of interest.

## Supporting information


Appendix S1

## Data Availability

The data that support the findings of this study are available from the corresponding author upon reasonable request.
