# Supplementary material for: Celecoxib attenuates hepatosteatosis by impairing de novo lipogenesis via Akt‐dependent lipogenic pathway
Source: J Cell Mol Med. 2022 Jun 17;26(14):3995–4006. doi: 10.1111/jcmm.17435 (PMC9279593; doi:10.1111/jcmm.17435)
Supplement: Supplementary file 1 — Appendix S1 [file JCMM-26-3995-s001.docx]

**Supplementary materials**

**Celecoxib attenuates hepatosteatosis by impairing *de novo* lipogenesis via Akt-dependent lipogenic pathway**

**Supplementary Figures**

**Figure S1.** Immunohistochemistry for total Akt (t-AKT) in liver samples from wild-type (WT) or AKT-injected mice. Positive staining (brown colored) broadly emerges in the livers of AKT-injected mice, indicating overexpression of Akt in the livers of mice subjected to hydrodynamic injection. CELE is the abbreviation of celecoxib. CELE-L and CELE-H represent intragastric administration of celecoxib at low (50 mg) and high (100 mg) doses, respectively.


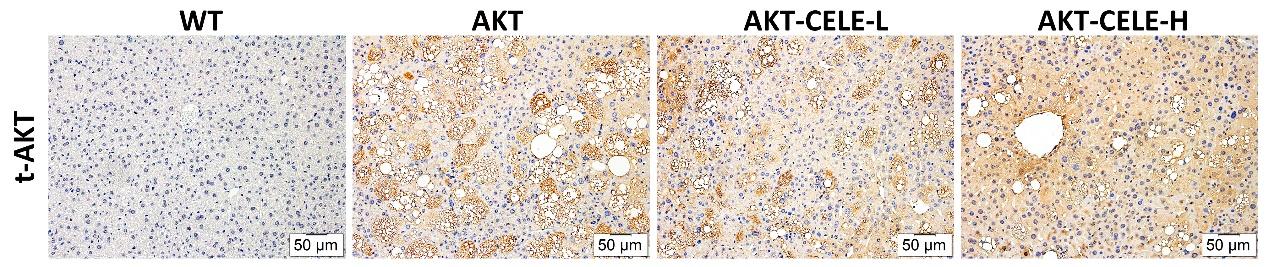


**Figure S2. Celecoxib reduces hepatic mRNA expression of** **representative lipogenic transcription factors and enzymes.** Histograms show the mRNA expression of key lipogenic transcription factors (*LXRα, ChREBP,* and *SREBP1*) (A-C) and enzymes (*FASN* and *ACC*) (D, E) quantified by the qPCR assay. Mean ± S.D., *n* = 5. ^#^*P* < 0.05, ^##^*P* < 0.01 and ^###^*P* < 0.001 *versus* the WT group; ^*^*P* < 0.05, ^**^*P* < 0.01, ^***^*P* < 0.001 *versus* the AKT group. CELE is the abbreviation of celecoxib. CELE-L and CELE-H represent intragastric administration of celecoxib at low (50 mg/kg) and high (100 mg/kg) doses, respectively.





**Supplementary Tables**

**Table S1.** List of the primary antibodies used for Western blot (WB) analysis and immunohistochemistry (IHC) analysis.

| **Protein** | **Antibody (and catalog number)** | **Application** |
| --- | --- | --- |
| ACC | Rabbit monoclonal (3676) | WB; IHC † |
| ChREBP | Rabbit monoclonal (13256-1-AP) | WB # |
| FASN | Rabbit monoclonal (3180) | WB; IHC † |
| HK2 | Rabbit monoclonal (2867) | WB † |
| IL-1β | Rabbit polyclonal (A19635) | WB $ |
| IL-6 | Rabbit polyclonal (GB11117) | WB * |
| LXRα | Rabbit monoclonal (14351-1-AP) | WB # |
| PGE2 | Rabbit monoclonal (ab2318) | IHC ^ |
| PKM2 | Rabbit polyclonal (7067) | WB † |
| Phospho-AKT (Thr308) | Rabbit monoclonal (13038) | WB † |
| Phospho-AKT (Ser473) | Rabbit monoclonal (4060) | WB † |
| Phospho-mTOR | Rabbit monoclonal (ab109268) | WB ^ |
| Phospho-RPS6 | Rabbit monoclonal (4858) | WB † |
| RPS6 | Rabbit monoclonal (14823-1-AP) | WB # |
| SREBP1 | Rabbit polyclonal (GB11524) | WB * |
| TNF-α | Rabbit polyclonal (17590-1-AP) | WB # |
| Total-AKT | Rabbit monoclonal (4691) | WB; IHC † |
| β-actin | Mouse monoclonal (A1978) | WB ^ |

† Provided by Cell Signaling Technology Inc. (Danvers, MA).

# Provided by Proteintech (Wuhan, China)

*Provided by Servicebio (Wuhan, China)

^Provided by Abcam (Cambridge, MA).

$ Provided by Abclonal (Wuhan, China)

**Table S2.** Sequences of primers used in quantitative real-time PCR (qPCR).

| **Gene** | **Sequences (5’ to 3’)** |
| --- | --- |
| *β-actin* | Forward: CGTTGACATCCGTAAAGACCTC  Reverse: TAGGAGCCAGGGCAGTAATCT |
| *LXRα* | Forward: GGGGTTGCTTTAGGGATAGG  Reverse: TTTCCGCTTTTGTGGACGA |
| *ChREBP* | Forward: TGCCATCTCCAGCCTCGT  Reverse: GCAGGTGGGATCTTGGTCTTAG |
| *SREBP1* | Forward: TGACCCGGCTATTCCGTGA  Reverse: CTGGGCTGAGCAATACAGTTC |
| *FASN* | Forward: CCAAGCAGGCACACACAA  Reverse: CACTCACACCCACCCAGA |
| *ACC* | Forward: GATGAACCATCTCCGTTGGC  Reverse: GACCCAATTATGAATCGGGAGTG |
